# Validity of transcutaneous bilirubin measurements during and after phototherapy in term and late preterm infants

**DOI:** 10.1007/s00431-024-05724-y

**Published:** 2024-09-13

**Authors:** Nirucha Thamwiriyakul, Pitiporn Siripattanapipong, Walaiporn Bowornkitiwong, Renoo Chaweerat, Sopapan Ngerncham

**Affiliations:** 1 Division of Neonatology, Department of Pediatrics, Faculty of Medicine Siriraj Hospital, Mahidol University, 2 Wanglang Road, Bangkoknoi, 10700 Bangkok Thailand; 2 Pediatric Nursing Division, Department of Nursing Siriraj Hospital, 2 Wanglang Road, Bangkoknoi, 10700 Bangkok Thailand

**Keywords:** Covered skin, Neonatal hyperbilirubinemia, Photo-opaque patch, Phototherapy, Transcutaneous bilirubin

## Abstract

The purpose of the study was to investigate correlation and concordance between total serum bilirubin (TSB) and transcutaneous bilirubin measured at covered (TcBC) and uncovered (TcBU) skin during and after discontinuation of phototherapy. A cross-sectional study included ≥ 34 weeks gestation infants requiring phototherapy for neonatal hyperbilirubinemia. In-house, photo-opaque patches were placed on infants’ sternums before phototherapy initiation. Simultaneous blood sampling for TSB, TcBC, and TcBU measurements were performed. Among 103 infants included in the final analysis, 70% were full-term. Covering skin during phototherapy resulted in strong TcBC-TSB correlation (*r* = 0.91, 95% CI 0.87–0.94, *P* < 0.001) compared to TcBU (*r* = 0.53, 95% CI 0.37–0.65, *P* < 0.001), persisting post-phototherapy (*r* = 0.88, 95% CI 0.82–0.91, *P* < 0.001). Bland–Altman analysis showed a higher mean difference and wider 95% limits of agreement for TcBU-TSB during phototherapy (-6.3 mg/dL and -11.1 to -1.6) vs TcBC-TSB (0.9 mg/dL and -1.2 to 2.9). Passing-Bablok regression analysis confirmed good agreement between TcBC and TSB.

*Conclusions*: The application of in-house, photo-opaque patches enhanced the correlation and agreement between TcBC and TSB during and after discontinuation of phototherapy. This may prove particularly useful in resource-limited settings where commercial devices are unavailable.

**Table:** 

**What is Known**:
• *Transcutaneous bilirubin measurement has been widely used as a screening method for neonatal hyperbilirubinemia.* • *The accuracy of transcutaneous bilirubin measurements during and after phototherapy in infants with hyperbilirubinemia has been debated.*
**What is New**:
• *Our study demonstrated that utilizing carefully designed photo-opaque patches enhanced the accuracy of transcutaneous bilirubin measurement during and after phototherapy.* • *Effective in-house alternatives are crucial in resource-limited settings where commercial opaque patches are not always accessible or affordable.*

**Supplementary Information:**

The online version contains supplementary material available at 10.1007/s00431-024-05724-y.

## Introduction

The American Academy of Pediatrics (AAP) recommends that all neonates exhibiting jaundice during birth hospitalization undergo bilirubin measurements, either of total serum bilirubin (TSB) or non-invasive transcutaneous bilirubin (TcB) [1]. While TcB measurement has shown reliable screening results for neonatal hyperbilirubinemia [2], its accuracy during and after discontinuation of phototherapy remains unclear [3]. Although the use of commercial photo-opaque patches has revealed better correlation between TcB and TSB during phototherapy [4, 5], limited accessibility in certain regions has prompted the development of an in-house alternative. This study aimed to investigate the correlation and concordance between TSB and TcB measurements taken on covered (TcBC) and uncovered (TcBU) skin during and after the discontinuation of phototherapy, using in-house, photo-opaque patches.

## Materials and methods

This cross-sectional study was conducted at a high-risk nursery of a 2500-bed university hospital in Bangkok, Thailand. Infants with gestational age of at least 34 weeks, who exhibited TSB levels meeting the phototherapy threshold, were enrolled. Infants requiring exchange transfusion for their hyperbilirubinemia or with skin disorders manifesting non-physiologic skin peeling were excluded.

The in-house, photo-opaque patches were developed by the investigators and the production steps are demonstrated in Supplementary Fig. 1. The patch was attached to the infant’s sternum prior to initiating phototherapy. This patch was then removed to expose the infant’s skin for TcBC measurements. TcBU measurements were obtained from an adjacent area of skin exposed to phototherapy. Bilirubin was measured as TSB, TcBC, and TcBU. All infants had a TSB drawn with every TcB measurement.

Treatment plans and bilirubin measurement schedules during and after discontinuation of phototherapy were determined by attending physicians following the guidelines of AAP [6] and Maisels et al. [7] for infants $\ge$ 35 and < 35 weeks gestation, respectively. Bilirubin measurements were typically scheduled every 12–24 h during phototherapy period and once at 12–24 h after its discontinuation. Blood samples drawn for TSB measurements were analyzed using the NEO-BIL Plus bilirubinometer (Das, Rome, Italy) in the hospital’s laboratory. Both TcBC and TcBU measurements were performed concomitantly by the same pediatric resident in the high-risk nursery using the JM-105 (Dräger, Lübeck, Germany). TcB measurements were conducted within a timeframe of 30 min before or after drawing blood for TSB. The researchers recorded the average of two consecutive TcB readings calculated by the JM-105 bilirubinometer. TSB and TcB measurements were independently performed by two personnel who were blinded to each other’s recorded values.

## Statistical analyses

Sample size calculation was based on a correlation between TSB and TcB of 0.74 derived from a previous study[8]. With a hypothesized correlation of 0.85, 80% statistical power, a significance level of 0.05, and 20% dropout rate, a sample size of 106 was required. Data were analyzed using Predictive Analytics Software (PASW) version 28.0 (SPSS Inc., Chicago, USA) and MedCalc version 17.2 (MedCalc Software, Ostend, Belgium). Pearson Correlation Coefficient (r) was calculated to assess the correlation between TSB and TcBC as well as TcBU. The concordance between TSB and TcBC as well as TcBU were examined using Bland–Altman analysis and Passing-Bablok regression analysis.

## Results

A total of 108 infants were enrolled in this study between December 2016 and March 2017. Five infants were excluded from the final analysis; three had their photo-opaque patches displaced during phototherapy, and two had parental consent withdrawn.

Demographic and clinical characteristics of all included infants are shown in Table 1. During phototherapy, 103 infants had their first bilirubin measurement, while 68 had a second measurement. After phototherapy discontinuation, bilirubin measurements were performed in 101 infants due to patch displacement in two infants. The timing at TSB measurement after the initiation and after discontinuation of phototherapy, the absolute time interval between TSB and TcB measurement, as well as TSB values during and after discontinuation of phototherapy are presented in Supplementary Table 1. The median total duration of patch attachment was 62 h, ranging from 24 to 90 h.

**Table 1:** Demographic and clinical characteristics of 103 infants included in the final analysis

Characteristics	Values
Maternal age (y), mean ± SD	29.2 ± 6.1
Male, n (%)	60 (58)
Full term infant, n (%)	72 (70)
Gestational age (wk), mean ± SD	37.3 ± 1.6
Birth weight (g), mean ± SD	2,950 ± 481
Total serum bilirubin before phototherapy initiation (mg/dL), mean ± SD	13 ± 2.7
Age of infant at the start of phototherapy (h), mean ± SD	63.2 ± 24.3
Duration of phototherapy (h), mean ± SD	35.2 ± 12.1
Causes of hyperbilirubinemia, n (%) • ABO incompatibility • G6PD deficiency • Suboptimal breastfeeding • Subgaleal/cephalhematoma • Jaundice of prematurity • Inconclusive	22 (21.4) 8 (7.8) 33 (32.0) 4 (3.9) 11 (10.7) 25 (24.3)

**Fig. 1:**
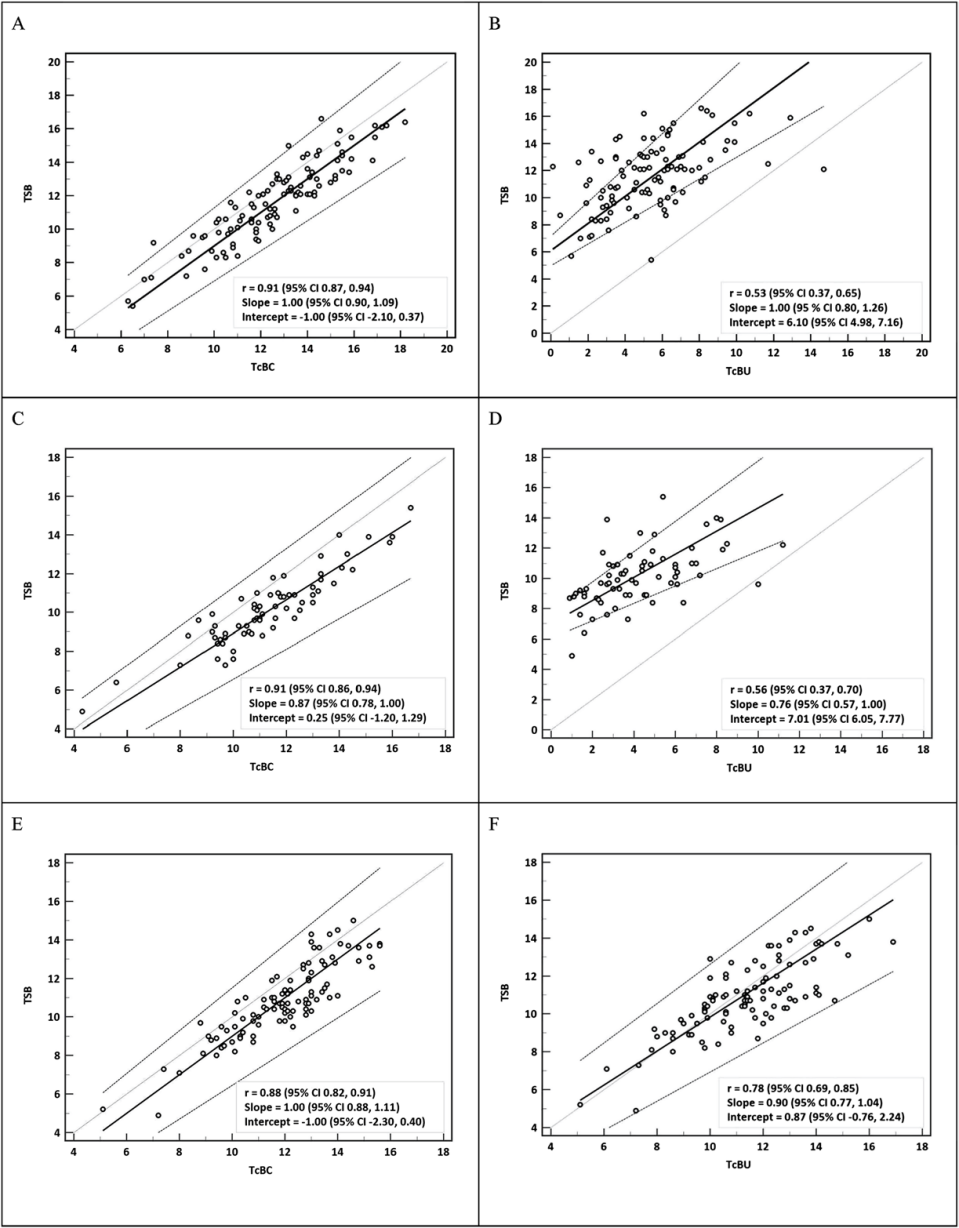
Scatter plots with Passing-Bablok regression lines (thick black line) along with 95% confidence interval (dashed lines) and identity lines (thin grey line) for bilirubin levels measured by two methods, total serum bilirubin (TSB) and transcutaneous bilirubin taken on covered (TcBC) and uncovered (TcBU) skin. Scatter plots between TSB and TcBC during the first (**A**) and second (**C**) measurement during phototherapy and after discontinuation of phototherapy (**E**). Scatter plots between TSB and TcBU during the first (**B**) and second (**D**) measurements during phototherapy and after discontinuation from phototherapy (**F**). Their respective correlation coefficient (95% confidence interval) and Passing-Bablok regression analyses (slope and intercept) are presented

The scatter plots of TSB against TcB to demonstrate the correlation between TSB and TcB (both TcBC and TcBU), as well as Passing-Bablok regression analysis of the two methods of bilirubin measurement at three time points, are presented in Fig. 1. The high correlation between TSB and TcBC was consistent throughout the phototherapy period and remained after its discontinuation. The correlation between TSB and TcBU was moderate during phototherapy and increased after its discontinuation. The correlations of all comparisons shown in Fig. 1 were statistically significant. Bland–Altman analysis of the difference between TcB and TSB plotted against the mean of the two methods is shown in Supplementary Fig. 2. Mean differences between TcBC and TSB measurements were consistently smaller than those between TcBU and TSB at the same time points during phototherapy. TcBC slightly overestimated TSB during and after the discontinuation of phototherapy. However, the limits of agreement (LoA) for the difference between TcBC and TSB measurements at all time points revealed no significant systematic bias between the methods. On the other hand, TcBU underestimated TSB during phototherapy and the LoA for the difference between TcBU and TSB measurements during phototherapy was wider but improved after the discontinuation of phototherapy for 12–24 h. Significant systematic bias between TcBU and TSB measurements was demonstrated during, but not after, the discontinuation of phototherapy. Bland–Altman plots and Passing-Bablok regression analyses confirmed the concordance between TcBC and TSB measurements.

Skin erythema (*n* = 2) and superficial skin abrasions (*n* = 2) were observed in four neonates after patch removal and resolved within a few days after applying a topical ointment.

## Discussion

The implementation of our in-house photo-opaque patches during phototherapy effectively enhanced the performance of TcB measurements. This study demonstrated a strong correlation between TcBC and TSB during phototherapy, with both Bland–Altman and Passing-Bablok regression analyses confirming the concordance between the two measurements. However, it is noteworthy that the strong correlation and concordance between TcBU and TSB were only evident 12–24 h after discontinuation of phototherapy.

The LoA between TcBC and TSB, both during and after the discontinuation of phototherapy, were within ± 3 mg/dL, which is considered a clinically acceptable range [1, 6]. Prior studies using commercial patches reported variable findings [4, 5, 9], possibly due to differences in mean TSB among studies. TcB typically exhibits good reliability when TSB levels are < 15 mg/dL [1, 6, 10, 11]. The mean TSB in our study, as well as in the studies by Radfar et al. from Iran [5] and Zecca et al. from Italy [9], were below this threshold and revealed a strong correlation and good agreement between TcBC and TSB. In contrast, the mean TSB in Murli et al. [4]’s study from India was notably higher, and they found poor agreement between TcBC and TSB. The differing results between studies may also be partly attributable to variations in melanin levels [1] and different bilirubin rates of rise [12] across ethnicities. Aforementioned studies also measured TcB at different sites (forehead [5, 9] vs sternum [4]). Our study chose sternum due to the reduced likelihood of patch displacement because of its relatively flat and spacious surface as well as it being the preferred site for the JM-105 bilirubinometer. However, TcB measurements at the sternum and forehead have comparable correlations with TSB [8, 11]. We do not recommend the use of TcBU during phototherapy due to its low correlation with TSB and the presence of systematic bias. TcBU significantly underestimated TSB, which could lead to the premature discontinuation of phototherapy, potentially harming the infants. However, TcBU may be considered useful 12–24 h after the discontinuation of phototherapy. By implementing TcBC during phototherapy in our unit, we were able to reduce the frequency of blood draws, minimizing discomfort for infants. TSB was obtained only when TcBC was > 15 mg/dL [1] or rising compared to previous TcBC measurement. The affordability of our in-house photo-opaque patches, at approximately 0.3 Euro each, further underscores their utility, especially in resource-limited settings.

We excluded infants with a gestational age of < 34 weeks due to concerns about skin’s fragility, which may limit the generalizability of our results to this population. De Luca, et al. [13] measured TcBC in an area of skin already protected from light by part of the continuous positive airway pressure device in extremely preterm infants and revealed good correlation and agreement between TcBC and TSB. Additionally, the relatively low bilirubin level in our study raises questions about the applicability of our in-house patches in infants with higher bilirubin levels, warranting further assessment. Lastly, when using TcB measurements, specific TcB nomograms for designation of risk zones should be used appropriately, as the bilirubin rate of rise differs across ethnicities [12].

## Conclusion

This study demonstrated through well-designed, in-house, photo-opaque patches that TcB measurements on covered skin acted as a valuable method for full-term and late preterm infants during and after discontinuation of phototherapy. This may prove particularly useful in resource-limited settings where commercial devices are either unavailable or unaffordable.

## Supplementary Information

Below is the link to the electronic supplementary material.

Supplementary file1 Supplementary Figure 1. In-house photo-opaque patch development process. The photo-opaque patches had two main components, the envelope and patch. The envelope was made from black poster paper (130 g/m2) with 2.5 by 5.5 cm dimensions folded into 2.5 by 2.5 cm dimensions. This envelope was then securely attached to the non-adhesive side of a piece of Tegaderm® using thin double-sided tape. A circular hole, measuring 1.5 cm in diameter, was made in the center of the envelope and the Tegaderm® to serve as the measuring site for TcB. The patch was made from black cardboard (270 g/m2) with 2.5 by 2.8 cm dimensions. To enhance its photo-opaque properties, this patch was covered with food-grade aluminum foil. The patch was subsequently inserted between the two layers of the envelope. (JPG 561 KB)

Supplementary file2 Supplementary Figure 2. Bland-Altman analysis of the difference between transcutaneous bilirubin (TcB) and total serum bilirubin (TSB) plotted against the mean of the two measurements. Solid horizontal lines present the mean differences with error bars presenting 95% confidence interval. Dashed lines present the limits of agreement. Bland-Altman plots for comparison of TSB and TcB measured at covered skin (TcBC) during phototherapy-first measurement (A) and second measurement (C), and after phototherapy discontinuation (E); TSB and TcB measured at uncovered skin (TcBU) during phototherapy-first measurement (B) and second measurement (D), and after phototherapy discontinuation (F). (JPG 500 KB)

Supplementary file3 (DOCX 16.5 KB)

## Data Availability

Data are available from the corresponding author upon reasonable request.

## Abbreviations

AAP: The American Academy of Pediatrics
CI: Confidence interval
IQR: Interquartile range
Max: Maximum
Min: Minimum
SD: Standard deviation
TcB: Transcutaneous bilirubin
TcBC: Transcutaneous bilirubin measured at covered skin
TcBU: Transcutaneous bilirubin measured at uncovered skin
TSB: Total serum bilirubin
LoA: Limits of agreement
