# Supplementary figures and images for: Validity of transcutaneous bilirubin measurements during and after phototherapy in term and late preterm infants
Source: Eur J Pediatr. 2024 Sep 13;183(11):5037–41. doi: 10.1007/s00431-024-05724-y (PMC11473562; doi:10.1007/s00431-024-05724-y)

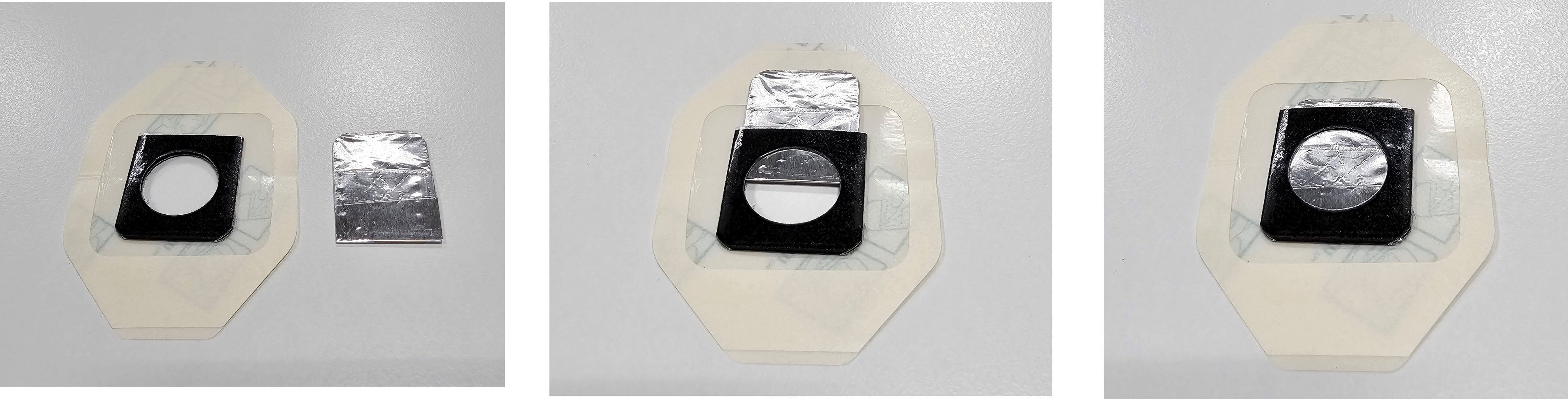

Supplement: Supplementary file 1 — Supplementary file1 Supplementary Figure 1. In-house photo-opaque patch development process. The photo-opaque patches had two main components, the envelope and patch. The envelope was made from black poster paper (130 g/m2) with 2.5 by 5.5 cm dimensions folded into 2.5 by 2.5 cm dimensions. This envelope was then securely attached to the non-adhesive side of a piece of Tegaderm® using thin double-sided tape. A circular hole, measuring 1.5 cm in diameter, was made in the center of the envelope and the Tegaderm® to serve as the measuring site for TcB. The patch was made from black cardboard (270 g/m2) with 2.5 by 2.8 cm dimensions. To enhance its photo-opaque properties, this patch was covered with food-grade aluminum foil. The patch was subsequently inserted between the two layers of the envelope. (JPG 561 KB) [file 431_2024_5724_MOESM1_ESM.jpg]

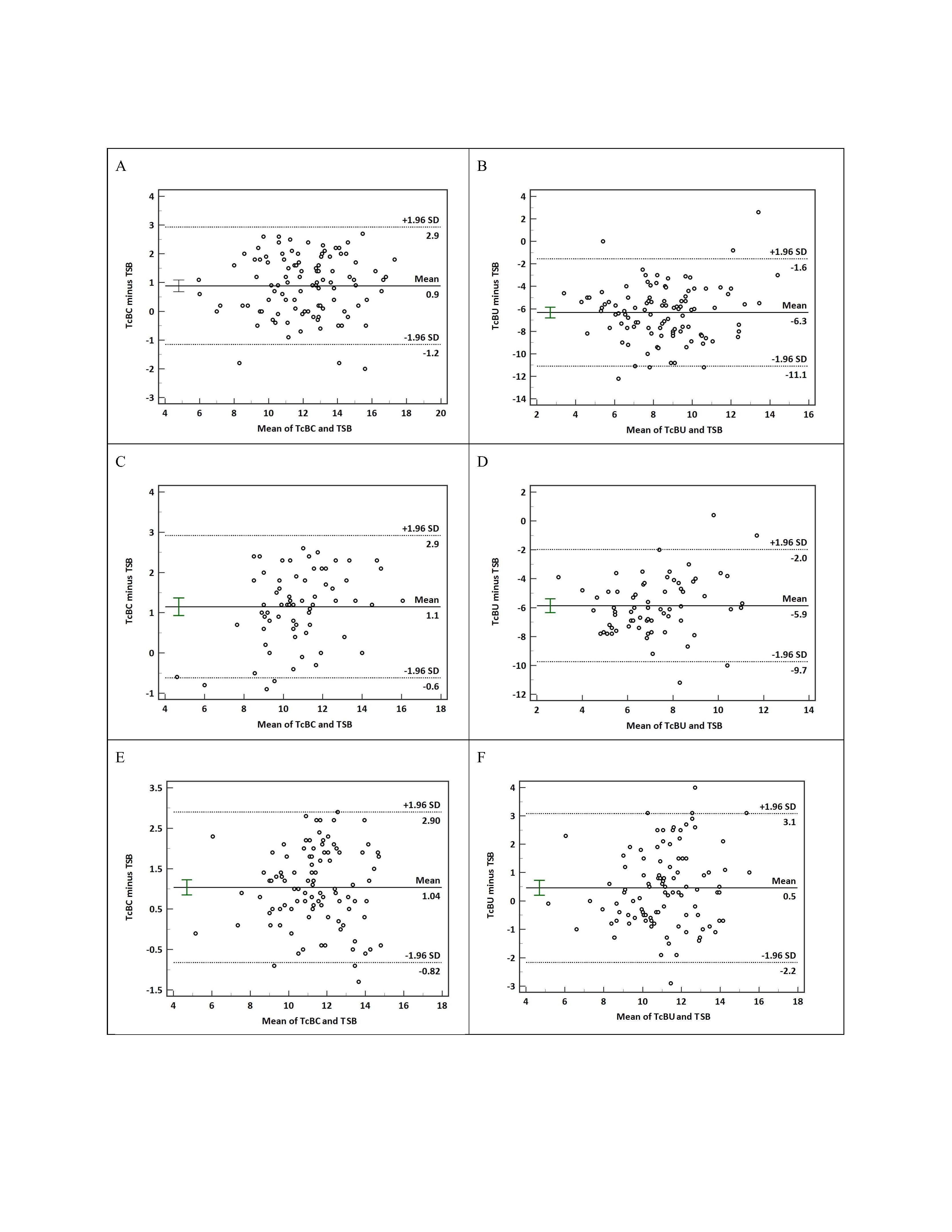

Supplement: Supplementary file 2 — Supplementary file2 Supplementary Figure 2. Bland-Altman analysis of the difference between transcutaneous bilirubin (TcB) and total serum bilirubin (TSB) plotted against the mean of the two measurements. Solid horizontal lines present the mean differences with error bars presenting 95% confidence interval. Dashed lines present the limits of agreement. Bland-Altman plots for comparison of TSB and TcB measured at covered skin (TcBC) during phototherapy-first measurement (A) and second measurement (C), and after phototherapy discontinuation (E); TSB and TcB measured at uncovered skin (TcBU) during phototherapy-first measurement (B) and second measurement (D), and after phototherapy discontinuation (F). (JPG 500 KB) [file 431_2024_5724_MOESM2_ESM.jpg]
