# Supplementary material for: Validity of transcutaneous bilirubin measurements during and after phototherapy in term and late preterm infants
Source: Eur J Pediatr. 2024 Sep 13;183(11):5037–41. doi: 10.1007/s00431-024-05724-y (PMC11473562; doi:10.1007/s00431-024-05724-y)
Supplement: Supplementary file 3 — Supplementary file3 (DOCX 16.5 KB) [file 431_2024_5724_MOESM3_ESM.docx]

**Supplementary Table 1.** Total serum bilirubin and transcutaneous bilirubin measurements during and after discontinuation of phototherapy.

|  | **During phototherapy** | | **After phototherapy discontinuation**  (n = 101)^†^ |
| --- | --- | --- | --- |
|  | ***1^st^ measurement***  (n = 103) | ***2^nd^ measurement***  (n = 68)^*^ |  |
| ***Time at TSB measurement (h)*** | | | |
| Median (IQR) | 20 (19-21)^‡^ | 44 (43-45)^‡^ | 20 (19-21)^§^ |
| ***Absolute time interval between TSB and TcB measurement (min)*** | | | |
| Median (IQR) | 10 (5-15) | 10 (5, 15) | 5 (5-8) |
| ***Bilirubin measurement (mg/dL); mean (SD)*** | | | |
| TSB | 11.7 (2.4) | 10.1 (1.9) | 10.9 (1.9) |
| TcBC | 12.6 (2.4) | 11.3 (2.2) | 11.9 (1.9) |
| TcBU | 5.3 (2.6) | 4.3 (2.3) | 11.3 (2.1) |
| ^*^The number of infants that continued phototherapy and had a second bilirubin measurement. ^†^Of the 103 infants underwent bilirubin measurements after discontinuation of phototherapy, two were excluded due to patch-displacement. ^‡^The interval between phototherapy initiation and bilirubin measurement. ^§^The interval between phototherapy discontinuation and bilirubin measurement.  Abbreviations: IQR, interquartile range; TcB, transcutaneous bilirubin; SD, standard deviation; TcBC, transcutaneous bilirubin measured at covered skin; TcBU, transcutaneous bilirubin measured at uncovered skin; TSB, total serum bilirubin. | | | |
